# Data Acquisition for Conservation Assessments: Is the Effort Worth It?

**DOI:** 10.1371/journal.pone.0059662

**Published:** 2013-03-26

**Authors:** Virgilio Hermoso, Mark J. Kennard, Simon Linke

**Affiliations:** Australian Rivers Institute and Tropical Rivers and Coastal Knowledge, National Environmental Research Program Northern Australia Hub, Griffith University, Nathan, Queensland, Australia; Swansea University, United Kingdom

## Abstract

When identifying conservation priorities, the accuracy of conservation assessments is constrained by the quality of data available. Despite previous efforts exploring how to deal with imperfect datasets, little is known about how data uncertainty translates into errors in conservation planning outcomes. Here, we evaluate the magnitude of commission and omission error, effectiveness and efficiency of conservation planning outcomes derived from three datasets with increasing data quality. We demonstrate that investing in data acquisition might not always be the best strategy as the magnitude of errors introduced by new sites/species can exceed the benefits gained. There was a trade-off between effectiveness and efficiency due to poorly sampled rare species. Given that data acquisition is limited by the high cost and time required, we recommend focusing on improving the quality of data for those species with the highest level of uncertainty (rare species) when acquiring new data.

## Introduction

Conservation planning has gained increasing attention from the scientific community and stakeholders as an essential way of aligning socio-economic development and conservation needs to secure the long-term persistence of biodiversity. Systematic conservation planning [1] represents an advance from ad-hoc conservation practices towards the implementation of efficient conservation management. This strategy leads to more cost-effective management recommendations by explicitly defining conservation objectives and integrating socio-economic (e.g., acquisition or stewardship cost) and other ecological (e.g., connectivity) aspects when looking for optimal allocation of priority areas for conservation.

The accuracy of conservation plans that arise from systematic planning depends on the quality of data on biodiversity patterns or other surrogates such as environmental classifications or habitat types available. Poor-quality or sparse data is potentially subject to high uncertainty and can lead to poor decision-making [2], the misuse of the limited resources available and ultimately the failure of conservation practice. Errors in conservation planning outputs associated with poor quality data can reduce effectiveness (e.g., when a species is erroneously thought to be present within a reserve, or commission errors) and efficiency (e.g., when a species is erroneously thought to be absent forcing the selection of additional and unnecessary areas, or omission errors). Despite the clear benefit of reducing uncertainties in conservation assessments, our capacity to make better informed decisions is constrained by the cost and time required to collect data [3], [4], [5], [6]. Conservation planners and stakeholders do not have access to complete information on biodiversity patterns (e.g., species distribution maps) and ecological processes aiming to be protected. Instead, conservation assessments are often carried out using sparse biological data or coarse surrogates such as habitat types obtained from remote sensing information [7]. Moreover, delaying conservation actions for improved knowledge on biodiversity patterns is not always the most appropriate strategy [6]. The effective protection of biodiversity might be compromised by habitat lost if the delay is too long. Understanding the limitations and consequences of uncertainties in input data is therefore a key issue in developing robust conservation recommendations from systematic planning [8]. Multiple efforts have been devoted to exploring the suitability of different types of data as surrogates for biodiversity patterns [9], [10], strategies to reduce uncertainties in the data [11], [12], [13] or how to explicitly account for those uncertainties in the planning process [14], [15], [16]. However, little is known yet about the link between the level of uncertainty in the input data and resulting errors in the conservation planning outcomes [17]. For example, we do not know the expected magnitude of commission and omission errors in conservation derived from a given level of uncertainty in the data. This makes it difficult to evaluate the relative risk taken when using poor quality data. Answering these types of questions should then be a priority to increase reliability of systematic conservation planning [8], [18] and help stakeholders evaluate the risks associated with imperfect data [14].

Here, we use a data-rich area in northern Australia to explore whether investing in new data acquisition is an adequate strategy to deal with errors in conservation planning outcomes derived from the use of poor datasets. Furthermore, we also explore if for any given dataset (either small or large) there is a significant improvement in conservation planning outcomes by constraining the data used to those species with low uncertainty. We model the spatial distribution of freshwater fish species using the entire dataset and use it as the true distribution of each species resembling the best available information [6], [8]. We then use three alternative distribution maps obtained from models built on different subsets of the database to evaluate the effect of data availability on conservation planning outcomes. We assume these maps to represent the information that a stakeholder would have available if the area had not been extensively surveyed. We also test the effect of constraining the data used in the planning process to species with low uncertainty levels by running independent analyses for different subsets of species for each model. We then evaluate performance through measures of commission and omission errors, effectiveness (proportion of species that are adequately represented) and efficiency (ratio species representation/cost in terms of number of planning units required). We use this case study to demonstrate the trade-offs associated with the use of poor-quality data (and prone to errors) vs. more risk averse (but more expensive) options derived from data acquisition. This would help evaluate the risk associated with the use of poor quality data and better inform the need for new data acquisition.

## Methods

### Spatial Framework, Fish and Environmental Data

The study area spans across northern Australia’s rivers from the Fitzroy River in the Kimberley regions eastwards to the Jardine River in Cape York Peninsula. We sourced presence-absence data for 104 freshwater fish species across the study area from the Northern Australian Freshwater Fish Atlas (www.jcu.edu.au/actfr) updated by [19]. This dataset contains records for more than 2300 sampling sites, although we retained for further analysis only sites with true presence-absence data (n = 714 sites). For subsequent modelling purposes we translated these presence-absence records into a network of predictive units. We delineated 11508 subcatchments (102.7 km^2^ on average) using ArcHydro [20] for ArcGIS 9.3 [21] from a 9 second digital elevation model [22]. There were a total of 498 subcatchments containing at least one sampling site. For those subcatchments with more than one record (n = 216) we combined the list of all species reported to produce a single record. We discarded from the dataset all the species with less than five occurrences, due to difficulties in modelling the distribution of these extremely rare species and the potential bias they would introduce to the analyses. Our final dataset comprised 70 fish species with an average frequency of occurrence of 95 subcatchments (range 5–433). Alternative surrogates of biodiversity patterns are commonly used in conservation planning, such as environmental classifications or habitat/vegetation types (known as coarse-filter surrogates). We focused on evaluating predictive models and do not compare our results against coarse-filter surrogates approaches as priority areas identified using these types of surrogates might not represent biodiversity better than random unless the classification clearly reflects the biodiversity patterns that they aim to represent or substitute [23]. In addition, previous studies highlighted the poor performance of coarse-filter surrogates at representing freshwater fish assemblages (e.g., [24]).

**Figure 1:**
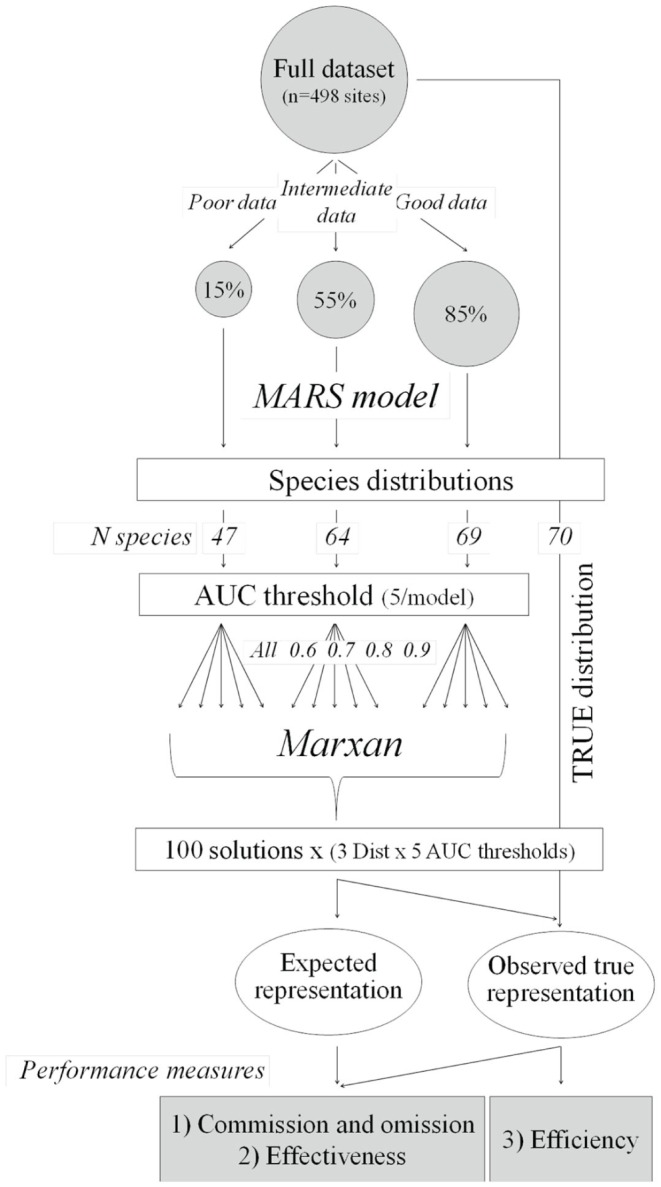
Flow diagram of analysis. We built four different predictive models: a) on the complete dataset (true distribution model) and b) three subsets of that dataset to simulate different data availability scenarios. We used the model outputs from the incomplete datasets to identify priority areas for conservation (using Marxan software package). In order to test the effect of species uncertainty on conservation outcomes, we ran independent analyses for different subsets of species, using the Area Under the ROC Curve (AUC) to filter species with increasing certainty. Results from Marxan were compared against the true distribution to obtain estimates of three different performance measures: 1) commission and omission errors, 2) effectiveness, and 3) efficiency.

An outline of the overall process we used to evaluate the role of data availability on conservation planning outcomes is provided in Figure 1. We built four different predictive models: a) on the complete dataset (true distribution model) and b) three subsets of that dataset to simulate different data availability scenarios using the same set of predictive variables and modelling technique. We used the model outputs from the incomplete datasets to identify priority areas for conservation (using Marxan software package) using the different species distributions as surrogates for biodiversity patterns. In order to test the effect of species uncertainty on conservation outcomes, we ran independent analyses for different subsets of species, using the Area Under the ROC Curve (AUC) to filter species with increasing certainty (higher AUC values). Results from Marxan were compared against the true distribution to obtain estimates of three different performance measures: 1) commission and omission errors, 2) effectiveness, and 3) efficiency.

### Predictive Modelling of Species Distributions

Nine ecologically-relevant landscape-scale environmental variables were selected from a larger number of candidate variables for use in the predictive models which were derived from the National Environmental Stream Attributes database for rivers [22]. We used Principal Component Analysis (PCA) to select a set of nine non-redundant environmental attributes that explain a high proportion of the environmental variability in the study area [25] (Table S1).

We used Multivariate Adaptive Regression Splines (MARS, [26]) to model the spatial occurrence of the 70 fish species. MARS is a method of flexible non-parametric regression modelling [27] useful for modelling complex non-linear relationships between response and explanatory variables. The model was built on the whole dataset (n = 498 subcatchments). Model accuracy was evaluated using two complementary approaches: deviance explained and the area under the receiver operating characteristic curve (ROC, [28]). The area under the ROC curve (AUC) was assessed through a k-fold cross validation procedure [29]. In this process the data set is randomly divided into k exclusive subsets and model performance is calculated by successively removing each subset, re-fitting the model with the remaining data, and predicting the omitted data. The average error when predicting occurrence in new sites can then be calculated by averaging the AUC across each of the subsets [26]. Deviance complements AUC because it expresses the magnitude of the deviations of the fitted values from the observations. We retained these measures as an estimate of the uncertainty around the predictions for each species.

The model was then used to predict the probability of occurrence of each species in all the unsurveyed subcatchments. Probabilities of occurrence were transformed into presence-absence data for posterior analyses using the optimal threshold obtained from the cost method in the presence-absence package in R [30]. This method finds an optimal threshold for each species that balances the relative cost of false positive and false negative predictions [28]. Given that these predictions were made with the best and more accurate dataset available, we will treat them as our true species distribution for subsequent analyses (see [6], [17] for similar approach).

### Data Availability and Uncertainty Scenarios

We built three additional species distribution models to simulate the effect of data availability on model errors (Fig. 1). With these models we intended to represent the data that would be available for stakeholders in data-poor areas [17]. We started using a random subset of 15% of the data (n = 75 subcatchments, hereafter termed “poor data model”), and added new data randomly selected from the set of subcatchments not included yet up to complete 55% (intermediate data model) and 85% (good data model) of the total available (n = 274 and 423 subcatchments respectively). We used the same set of environmental predictors across all models (same set of variables used for constructing the true model above, Table S1). We applied the same minimum threshold of 5 occurrences for a species to be included in the predictive model developed for each dataset. This resulted in 47, 64 and 69 species modelled using the poor, intermediate and good data model respectively (Fig. 1). We then used each model to predict the spatial distribution of species under these three data-constrained scenarios and calculated the optimal threshold to transform the probabilities of occurrence into presence-absence data. We measured the rate of false positive occurrences (1- proportion of correctly predicted presences) and false negative occurrences (non-predicted presences) for each of these three models by comparing the predicted distribution under each data constraint scenario to the true distribution.

### Identification of Priority Areas for Conservation

We used the predictions from each model as surrogates of biodiversity patterns to identify priority areas for conservation. We used the software Marxan [31] to find an optimal set of planning units to represent at least 10% of each species’ predicted distribution at the minimum cost. Given our special interest in evaluating the effect of different models’ outputs we used a constant cost for each planning unit, so our objective translated into finding the minimum set of planning units to achieve the conservation targets [32]. Although we acknowledge that the use of economic cost enhances the efficiency of recommendations delivered by systematic conservation planning, we wanted to isolate the effect of data availability from other issues. Although this assumption entails a simplified planning environment it will help our findings to be applicable to a wider range of circumstances.

To further explore the effect of including species with different uncertainty levels in the analyses we repeated the selection of priority areas for different subset of species for each predictive model. We constrained the optimization process to species with an AUC>0.6, 0.7, 0.8 and 0.9 for these new scenarios (AUC threshold scenarios hereafter). Therefore, we ran a total of 15 different scenarios (3 models×5 AUC thresholds). For each of them we retained 100 solutions obtained after 1.5 M iterations each for further analyses.

To rule out potential bias in the results due to the different number of species included in the analyses for the data availability strategy, we compared the results when using all the species modelled and only the ones common to all models (n = 47 species) across 100 solutions from Marxan. With this aim we ran Marxan for the whole set of species modelled and constraining the analyses to the species common to all models for each of the predictive models. So two different set of results were obtained for each data availability scenario, including all modelled species and considering only the species common to all models. If there were significant differences in the performance measures detailed above between both approaches we would use the set of species common to all the models only for subsequent analyses and avoid in this way the bias introduced by new species added to analyses and allow for comparisons across models.

### Commission and Omission Errors

We measured the commission and omission error rates for each solution obtained from Marxan (1500 solutions, 100 solutions×15 scenarios) by comparing the expected and observed representation achieved for each species. The expected representation was measured as the number of occurrences within solutions according to the surrogate data used in the optimization process (each of the three different predictive models). This was treated as the expected representation since it resembles the potential representation that would be achieved if the predictions used had no associated errors. The observed representation was measured as the number of occurrences within solutions according to the true spatial distribution. We then measured the rate of commission and omission errors as the proportion of expected representation that was not truly achieved (Equation 1).
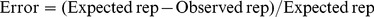
(1)


Whenever the expected and observed representations are similar, the error obtained from Equation 1 is close to 0, indicating low commission or omission error. However, when the expected representation is higher or lower than the observed representation, the error value will depart from 0 and be negative or positive, indicating commission and omission errors, respectively.

Systematic conservation planning aims to informing conservation decision-making on cost-effective priorities rather than providing a conservation plan to be implemented. For this reason, our evaluations on errors are constrained to the recommendations that would be offered to stakeholders rather than errors in the final implementation of conservation plans.

### Effectiveness and Efficiency

We measured the effectiveness of each solution as the proportion of the species that truly achieved the target (observed representation ≥ target). Given that targets were defined according to each predictive model’s output as 10% of the expected distribution (model-specific target) they might also be exposed to error. For example, if a species’ distribution was significantly underestimated under any of the predictive models, its target would also be underestimated and so increase the likelihood of true underrepresentation in solutions. In order to estimate the effect of model errors on target setting, we measured the proportion of species that would achieve a target of 10% of their true distribution (observed representation ≥10% true distribution, or true target). We then compared whether each species had achieved the model-specific target but had not achieved the true target (labelled as a false positive target achievement) or vice versa (labelled as false negative target achievement). We also checked the number of species that did not achieve either the target or the true target.

Finally, we measured the efficiency of each solution as the average ratio across all species between the true representation and the total number of planning units required.

### Determinants of Commission and Omission Error Rates

We explored the importance of a set of factors potentially driving the observed commission and omission error rate. With this aim, we built a Generalised Linear Model (GLM) using a normal distribution and a log link function with the commission and omission error rate as dependent variable and seven different factors we wanted to test as independent variables. These included the rate of false positive and negative prediction errors, the model used to obtain the species’ distribution (poor, intermediate and good data models), the AUC and deviance of each species in each model, the AUC threshold used and each species’ prevalence in the dataset used for building each model. We used a forward stepwise variable selection procedure with a p<0.05 entry criterion to obtain the best model. We retained the adjusted R^2^ as an indicator of the model fit and each independent variables’ *Beta* coefficient and *P* value in the model as an estimate of their relative importance at explaining the dependent variable. The magnitude of the *Beta* coefficient allows comparing the relative contribution of each independent variable and the *P* value informs whether the effect was found to be significant or not. We would expect important factors to be included in a model that explains a high proportion of the dependent variable’s variance (high adjusted R^2^), with a high *Beta* coefficient. We tested independent variables for redundancy prior to analyses and included all the factors cited previously except the deviance explained since it was highly redundant with AUC (Pearson’s R = 0.73, while R<0.15 for the remaining pair wise correlations).

### Effect of Strategies to Improve Conservation Planning Outcomes

We used factorial ANOVA to test for significant changes in commission and omission error, effectiveness and efficiency when following the two alternative strategies evaluated here (increasing the amount of data used for the predictive models and constraining the analyses to species with low uncertainty –measured by the AUC). We included each strategy (e.g., model and AUC threshold) and their interaction as factors. In order to evaluate the net effect of the new species added when increasing the dataset in the overall commission and omission error rate, effectiveness and efficiency, we also used ANOVA to test for significant differences between results obtained using all the species modelled and for the subset of species common to all models.

## Results

### Species Distribution Predictive Models

The predictive model built on the whole dataset was good as the indicated by average AUC and explained deviance measures (Table S2), similar to model performance reported in previous applications of MARS predictive models [25], [33]. Both AUC and deviance explained increased from the poor-data to the high-data models (Table S2). As an indication of the improvement gained when adding new data for model construction, the proportion of species with AUC>0.9 rose from 2%, to 19% and 26% from the poor to the intermediate and high-data models, respectively. Similarly, the proportion of species with AUC>0.8 increased from 30% to 50% and 59% for the same models, respectively. This net improvement in modelling performance also translated into a reduction of errors in predictions. The rate of false positive occurrences decreased from 0.32 to 0.09 from the poor to the high-data model for the set of species common to both models. This decrease in false positive occurrences was also true for the set of species added in the intermediate and poor-data models, although the values where always lower than for the species common to all models (Table S2). The rate of false negative occurrences decayed even more abruptly when adding new data, for both set of species (common to all models and new additions; Table S2).

### Commission/Omission Errors

The inclusion of the new species in the planning process when more data were available for the models showed no major effect on commission and omission errors. Commission and omission errors for solutions where only species common to all models were used and when al modelled species (including new additions of rare species) were very similar (Pearson’s R^2^ = 0.99 in all cases). For this reason we hereafter use the solutions where all the modelled species had been included.

**Figure 2:**
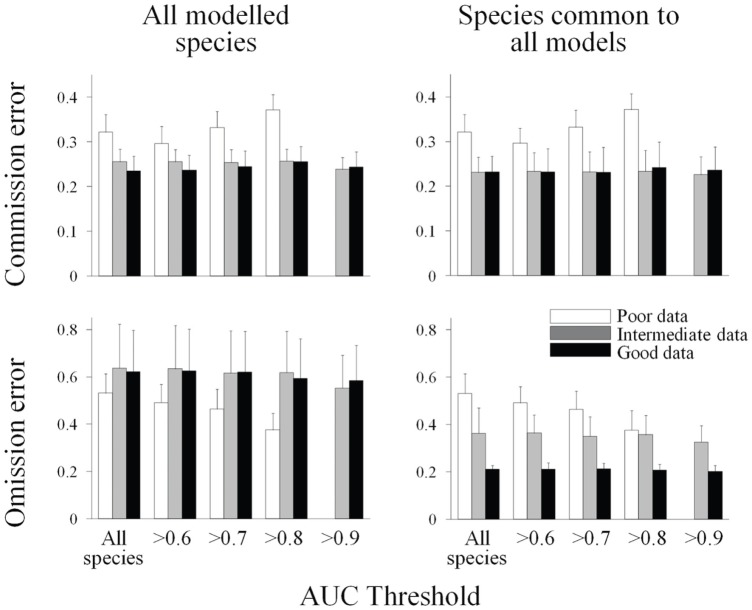
Change in commission and omission errors across different scenarios. Effect of the two different strategies (increase of data available for model construction and use of increasing AUC threshold) to reduce commission and omission error tested in this manuscript. Average and standard error values across all species included in the model (note that increasing set of species could be modelled when adding new data; n = 47, 64 and 69 species for the poor, intermediate and good data models respectively) and only for species common to all models (n = 47 species).

The rate of false positive and negative occurrences were the most important determinants of commission and omission errors explored in the GLM model (Table 1). This model also revealed the amount of data used in model and the AUC of each species as additional important factors explaining but at a significant distance from the rate of false positive and negative occurrences. The threshold applied to the AUC appeared in the final model but with a non-significant effect and the species’ prevalence was not selected (Table 1). These results were supported and refined by the ANOVA analysis on the effect of the two alternative strategies tested here (increasing the amount of data used for the predictive models and constraining the analyses to species with low uncertainty) on variation in commission and omission errors. There was a significant decrease in omission errors across models (Table S3) but not in commission errors (Table S3; Fig. 2). On the other hand, there were not significant differences either in commission nor omission errors across AUC thresholds (Table S3; Fig. 2). The interaction term in the factorial ANOVA was non-significant (Table S3). In all cases there were significantly higher errors for the newly added species in each model than for the remaining common to all models (Table S3; Fig. 2). In summary, expanding the dataset used for building the predictive models was the only strategy that significantly reduced omission errors. This was true only for the species common to all models (the ones that accumulated more presences when adding new data, since the average prevalence of species common to all models increased from 25 to 76 and 118 occurrences from the poor-data to the intermediate and high-data models respectively).

**Table 1:** Multiple regression model used for evaluating the relative importance of different factors as drivers of commission and omission errors.

Factor	Beta	t(828)	p-level	AdjR^2^
False negative occurrence rate	−0.82	−47.5	<0.001	0.79
False positive occurrence rate	−0.47	−25.6	<0.001	
Amount of data	−0.14	−7.0	<0.001	
AUC	−0.07	−4.3	<0.001	
AUC Threshold	0.02	1.3	0.205	
Intercept		27.9	<0.001	

The rate of false positive and negative occurrences was calculated by comparing the spatial distribution of species under each predictive model and the true distribution. Amount of data refers to each of the three different models tested (poor, intermediate and good quality data models), the Area Under the Curve (AUC) was measured for each species and model through a K-fold validation procedure. Standardised Beta coefficients, a t statistic (degrees of freedom between parentheses) and an associated p value are shown. The adjusted R^2^ is also given.

### Effectiveness

**Figure 3:**
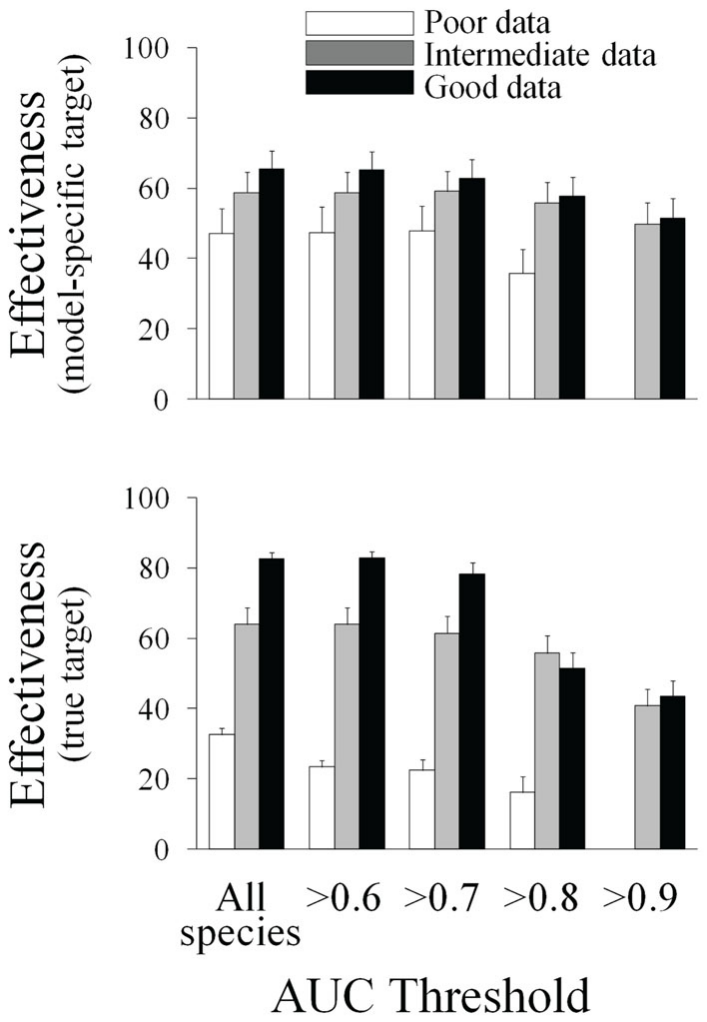
Change in effectiveness across different scenarios. Effect of the two alternative strategies tested on effectiveness measured as the proportion of species that achieve the model-specific target and true target. Average and standard error across 100 solutions obtained from Marxan are showed.

**Figure 4:**
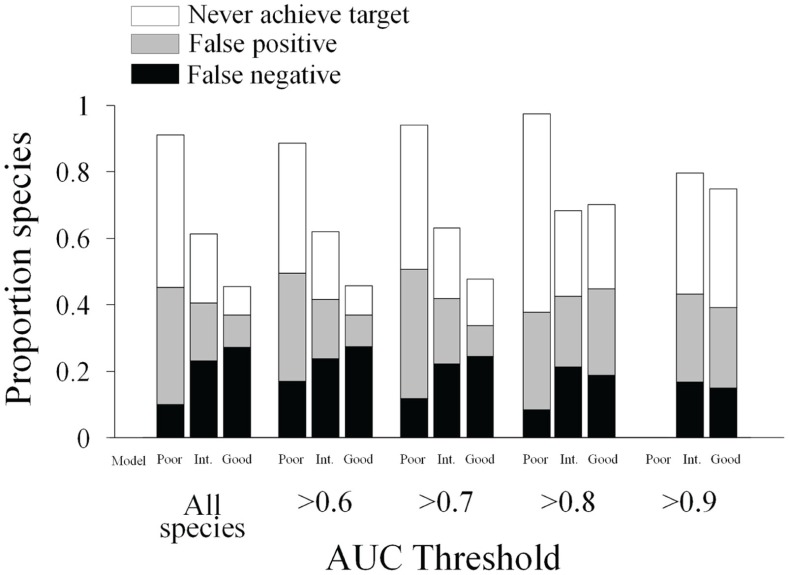
Change in false positive and negative errors across different scenarios. Proportion of species that did not achieve the neither the model-specific target nor the 10% of their true distribution (white); species that achieved the model-specific target but not their 10% true distribution (false positive in grey); and species that did not achieve the species-specific model but did achieve their 10% true distribution (false negative in black). Each bar shows the average values across 100 solutions obtained from Marxan for a given combination of model and AUC threshold.

The amount of data used for model construction always had significant effects on effectiveness in both types of targets, although stronger when testing differences in the true target (Table S3). On the other hand the AUC threshold strategy only had significant effects on effectiveness when attending to the true target (Table S3; Fig. 3). The interaction term in the factorial ANOVA (amount of data×AUC threshold) was significant for the true target effectiveness, while non-significant for the model-specific target (Table S3). The effect of errors in predictions did not only translate into commission and omission errors but biased target setting and the estimate of effectiveness. The rate of false positive target achievement decreased with data addition, while the rate of false negative increased with data addition (Fig. 4). Data addition proved to be beneficial at reducing the proportion of species that never achieved the target (either the true or model-specific targets). The use of different AUC thresholds had no major effect (Fig. 4).

### Efficiency

**Figure 5:**
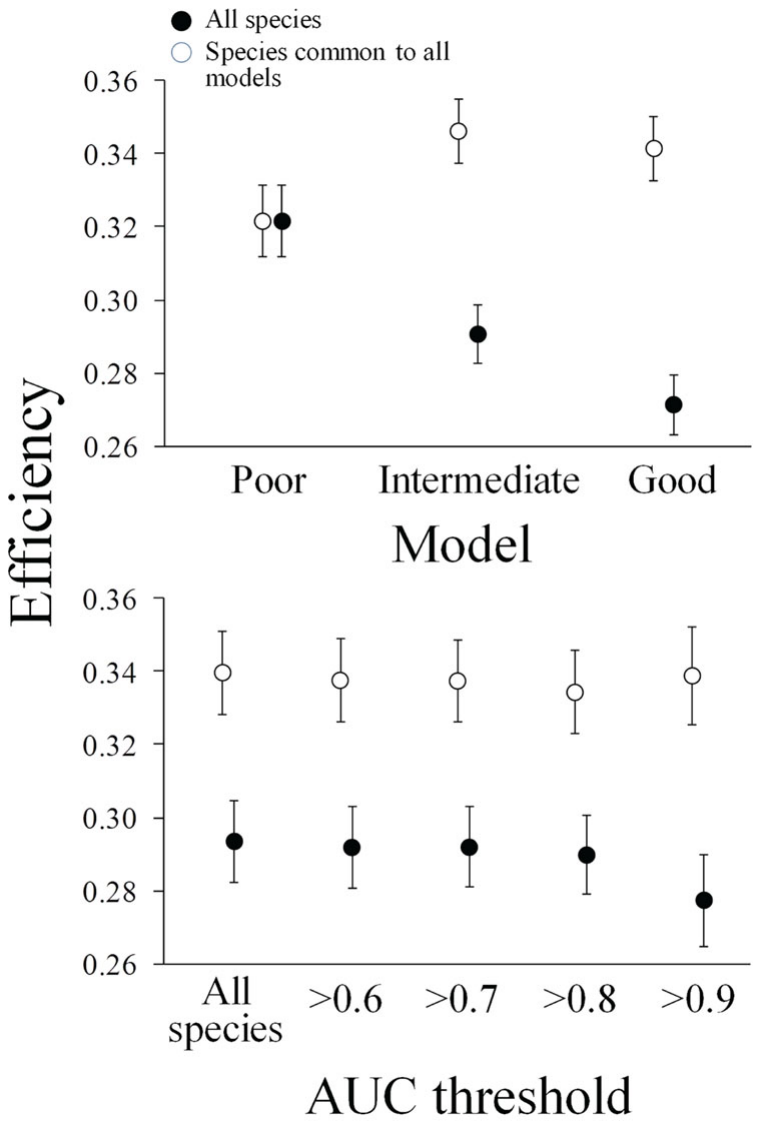
Change in efficiency across different scenarios. Effect of the two alternative strategies tested on efficiency measured as the ratio representation/number of planning units. Average and standard error across 100 solutions obtained from Marxan are showed.

There was a significant decrease in efficiency when adding new data to the predictive models (Table S3; Figure 5). However, there was a significant increase in efficiency when attending only to the species common to all models (Fig. 5), so we can conclude the decrease in efficiency was driven by the new species considered in the analyses when more data were available. On the other hand there were no significant differences when trying different AUC thresholds neither for all the species or the species common to all models (Table S3; Fig. 5). The interaction term in the factorial ANOVA was also non-significant in this case (Table S3).

## Discussion

Our results demonstrate that data acquisition might not always be the best strategy to increase the accuracy of conservation recommendations as the magnitude of the errors introduced by the new sites/species can exceed the benefits gained by reducing the errors for other species. These errors can reduce efficiency of solutions leading to the misuse of the limited resources available and ultimately the failure of conservation practice. There were trade-offs between the benefit at reducing representation errors and increasing efficiency mainly led by the influence of poorly sampled rare species. The value of biodiversity surveys has been highlighted as an effective way of increasing certainty in data [13], [34] and enhance the accuracy of conservation planning. However, our results align with other studies suggesting the value of reduced datasets. For example [35] reported that reserves identified using data from low sampling effort can be highly effective at representing species, even at their peak abundance areas. This does not disqualify the value of biodiversity surveys, given that it would be very difficult to detect some of the rarest species in the landscape (most in need of conservation) without intensive surveys. This may particularly apply in biogeographically complex or environmentally heterogeneous areas that may exhibit high species turnover and centres of endemism/rarity. Given that the addition of new high quality data is constrained by the high cost and time required, we would recommend concentrating survey efforts on gathering more data for those species with the highest uncertainties (especially rare species, see for example Gradsec in which sampling is focused on discrete areas selected to contain maximum environmental heterogeneity to minimize travelling between sites; [13], [36]) or incorporating these uncertainties explicitly in the decision-making process (e.g., information-gap theory; [16], [37], [38]). These would lead to a better informed decision-making and enhanced conservation practise.

### Commission and Omission Errors

Commission and omission errors were mainly associated with the rate of false negative and false positive occurrences in the distribution maps. As expected, commission errors were positively related to the rate of false positive occurrences, while omission errors were negatively related to the rate of false negative occurrences. These errors could be reduced by making more data available for the predictive models. However, while there was a continuous decrease in the rate of false negative errors and omission errors when adding new data, this decline was not so strong for false positive and commission errors. Given that reducing omission errors is a risk averse strategy in conservation planning [9], it would be reasonable to invest in further data acquisition even though the improvement in commission errors was not so pronounced. However, the benefit of this strategy was only true for species that were relatively common in the study area and that rapidly increased the number of presences in the dataset when adding new sampling sites. Data addition allowed some rare species initially excluded to be incorporated in the planning process as they fulfilled the threshold of minimum number of presences. However, the inclusion of these new species had a counterproductive effect as the magnitude of omission errors increased significantly to the point of veiling the benefit described above. There is thus a trade-off between the reduction of omission errors for common species and the increase in omission errors at the community level when rare species are considered.

To a lesser extent the rate of commission and omission error were related to the estimate of species-specific uncertainties obtained from the model validation process (AUC). There was not a high correlation between AUC and the rate of false positive and negative occurrences, which could indicate that some of the AUC values were overestimated (e.g., overfitting) or underestimated [39]. This could also explain the poor performance of the AUC-threshold strategy and may constrain the potential use of this estimate of species-specific uncertainty for approximating the potential risk associated with a given dataset for use in conservation planning. Given that our estimates of false positive and negative occurrences would not be available during the planning process further research is required to test alternative measures of species’ uncertainties that are more suitable for indicating the relative risk associated with a dataset (e.g., consensus analyses across different modelling techniques; although see [40]).

### Effectiveness and Efficiency

Our results show a second trade-off between effectiveness and efficiency for increasing amounts of data. There was a significant increase in effectiveness when adding new data as more species achieve the target [9], [35], [41]. The increase in effectiveness was specially marked when attending to the true target (more species achieved 10% of their true distribution). However, this increment in effectiveness was coupled with a decrease in efficiency. More species achieved the target, but at expenses of a significant reduction in efficiency. In our case, this reduction in efficiency was mainly due to the increment in omission errors associated with rare species described before. Due to inflated omission errors, more areas than needed were selected to adequately represent these rare species which caused a decline in efficiency in the overall conservation plan.

### Target Setting

Modelling errors (false positive and negative occurrences) also biased the target setting, which had not been explicitly addressed in conservation planning yet. Given that our model-specific targets were set as a proportion of the predicted spatial distribution, modelling mistakes translated into over or underestimated targets. This is not a trivial issue given that these targets could lead some species to be underrepresented (affecting reserves’ adequacy) or overrepresented (leading to bigger reserves than actually needed and then reducing efficiency). We demonstrate that the proportion of species that are erroneously thought to miss the target (and then not adequately represented) due to overestimation of targets can be reduced by adding new data. However, once again this gain might be neutralized by the increase in the proportion of species that are erroneously thought to achieve the target (more species are affected by underestimation of targets).

### Concluding Remarks

Our results clearly demonstrate that data acquisition is not always the best strategy to increase accuracy in conservation planning assessments. We highlight the value of sparse data as it might be suitable for portraying the spatial patterns of biodiversity surrogates used for conservation planning. Data addition led to an increase in effectiveness as more species were adequately represented within priority areas but at the expense of reducing efficiency. This strategy thus has a doubly pernicious economic effect on conservation planning: it is more expensive to produce conservation recommendations (increase in cost due to data collection) and these conservation recommendations are less efficient (more areas than needed are selected). Given that data acquisition is limited by the high cost and time required, we recommend focusing on improving the quality of data for those species with the highest level of uncertainty (rare species) when acquiring new data. Further studies are required to evaluate the suitability of different data acquisition strategies (environmentally driven strategies such as Gradsec mentioned above vs. the random addition we tested here) and to give a monetary value to the trade-offs showed here, so stakeholders could decide whether investing in new data acquisition is worth the effort.

## Supporting Information

Table S1 **Summary of average and standard error (SE) of model performance indicators across different species and models.** Results are presented separately for all the species common to all models (common in Table) and new species added when expanding the data set (new additions in Table). The performance of the true model is also showed.
(DOCX)

Table S2 **Summary of environmental attributes selected from the Principal Component Analysis carried out on the whole set of environmental variables available (which explained 72% of the total variance in the dataset).**
(DOCX)

Table S3 **F values & significant levels from ANOVA analyses for testing the effects of data addition, species uncertainty (AUC threshold) and their interaction on five different conservation planning performance measures.**
(DOCX)
