# Supplementary material for: Data Acquisition for Conservation Assessments: Is the Effort Worth It?
Source: PLoS One. 2013 Mar 26;8(3):e59662. doi: 10.1371/journal.pone.0059662 (PMC3608668; doi:10.1371/journal.pone.0059662)
Supplement: Table S1 — Summary of average and standard error (SE) of model performance indicators across different species and models. Results are presented separately for all the species common to all models (common in Table) and new species added when expanding the data set (new additions in Table). The performance of the true model is also showed. (DOCX) [file pone.0059662.s001.docx]

Table S1. Summary of average and standard error (SE) of model performance indicators across different species and models. Results are presented separately for all the species common to all models (common in Table) and new species added when expanding the data set (new additions in Table). The performance of the true model is also showed.

|  |  | Model | | | | | | | |
| --- | --- | --- | --- | --- | --- | --- | --- | --- | --- |
| Factor |  | Poor data model | | Intermediate data model | | Good data model | | True model | |
|  |  | Mean | SE | Mean | SE | Mean | SE | Mean | SE |
| False positive occurrences | common | 0.32 | 0.03 | 0.13 | 0.02 | 0.09 | 0.02 | - | - |
|  | new addition | - | - | 0.20 | 0.04 | 0.12 | 0.02 | - | - |
| False negative occurrences | common | 0.34 | 0.03 | 0.17 | 0.02 | 0.09 | 0.01 | - | - |
|  | new addition | - | - | 0.26 | 0.06 | 0.24 | 0.05 | - | - |
| Deviance | common | 0.20 | 0.02 | 0.28 | 0.02 | 0.27 | 0.02 | 0.32 | 0.02 |
|  | new addition |  |  | 0.43 | 0.04 | 0.41 | 0.03 |  |  |
| AUC | common | 0.73 | 0.02 | 0.80 | 0.01 | 0.80 | 0.01 | 0.83 | 0.02 |
|  | new addition | - | - | 0.84 | 0.02 | 0.86 | 0.02 | - | - |
