# Supplementary material for: Data Acquisition for Conservation Assessments: Is the Effort Worth It?
Source: PLoS One. 2013 Mar 26;8(3):e59662. doi: 10.1371/journal.pone.0059662 (PMC3608668; doi:10.1371/journal.pone.0059662)
Supplement: Table S2 — Summary of environmental attributes selected from the Principal Component Analysis carried out on the whole set of environmental variables available (which explained 72% of the total variance in the dataset). (DOCX) [file pone.0059662.s002.docx]

Table S2. Summary of environmental attributes selected from the Principal Component Analysis carried out on the whole set of environmental variables available (which explained 72% of the total variance in the dataset).

| Variable | Average | min | max | se |
| --- | --- | --- | --- | --- |
| Longitude at the pour point of the subcatchment | 134.66 | 122.15 | 145.36 | 0.06 |
| Stream and environs coldest month mean temperature | 12.31 | 6.79 | 20.80 | 0.02 |
| Catchment area divided at stream bifurcations | 1540.61 | 0.54 | 87009.70 | 59.65 |
| Catchment relief | 0.41 | 0.01 | 0.85 | <0.001 |
| Average slope of downstream flow path | 0.06 | 0.00 | 2.19 | <0.001 |
| Catchment average slope | 1.46 | 0.01 | 17.74 | 0.02 |
| Sub-catchment area | 56.11 | 0.07 | 4969.47 | 3.06 |
| Percentage tree cover | 66.40 | 0.00 | 100.00 | 0.39 |
| River Disturbance Index (Stein *et al.*2002) | 0.09 | 0.00 | 0.63 | <0.001 |

Reference:

Stein J.L., Stein J.A. & Nix H.A. (2002). Spatial analysis of anthropogenic river disturbance at regional and continental scales: identifying the wild rivers of Australia. Landscape Urban Plan., 60, 1-25.
