# Supplementary material for: Data Acquisition for Conservation Assessments: Is the Effort Worth It?
Source: PLoS One. 2013 Mar 26;8(3):e59662. doi: 10.1371/journal.pone.0059662 (PMC3608668; doi:10.1371/journal.pone.0059662)
Supplement: Table S3 — F values & significant levels from ANOVA analyses for testing the effects of data addition, species uncertainty (AUC threshold) and their interaction on five different conservation planning performance measures. (DOCX) [file pone.0059662.s003.docx]

Table S3. F values & significant levels from ANOVA analyses for testing the effects of data addition, species uncertainty (AUC threshold) and their interaction on five different conservation planning performance measures.

| Performance measure | Strategy to reduce errors in conservation planning outcomes | | |
| --- | --- | --- | --- |
|  | Data addition | AUC threshold | Data addition x AUC threshold |
| Commission errors | F_(2, 444)_= 1.4, P= 0.26 | F_(4, 397)_= 0.13, P= 0.39 | F_(7, 833)_= 0.27, P=0.96 |
| Omission errors | F_(2, 399)_= 31.6, P<0.001 | F_(4, 442)_= 0.13, P=0.97 | F_(7, 433)_= 0.13, P=0.99 |
| Effectiveness (model-specific target) | F_(2, 837)_= 39.7 P<0.001 | F_(4, 836)_= 10.7 P<0.001 | F_(7,833)_= 0.08 P=0.99 |
| Effectiveness (true target) | F_(2, 837)_= 119.8 P<0.001 | F_(4, 836)_= 0.74 P=0.53 | F_(7, 833)_= 2.6, P=0.01 |
| Efficiency | F_(2, 846)_= 7.15 P<0.001 | F_(4, 844)_= 0.6 P=0.62 | F_(7, 598)_= 0.5 P=0.85 |
